# Subjective memory complaints among older adults in Türkiye: prevalence and associated factors

**DOI:** 10.55730/1300-0144.6104

**Published:** 2025-09-22

**Authors:** Mustafa ÇETİN, Zehra SARIKAYA DEMİRBAŞ, Mehmet İlkin NAHARCI

**Affiliations:** Division of Geriatrics, Department of Internal Medicine, Gülhane Faculty of Medicine, University of Health Sciences, Ankara, Turkiye

**Keywords:** Subjective memory complaints, older adults, education level

## Abstract

**Background/aim:**

Subjective memory complaints (SMCs), defined as self-perceived declines in memory performance, are common among older adults and may serve as early indicators of neurocognitive impairment. Despite their clinical relevance, no previous studies have examined the prevalence and associated factors of SMCs in Türkiye.

**Materials and methods:**

A total of 500 community-dwelling older adults were included in this retrospective, cross-sectional, observational study; all underwent a comprehensive geriatric assessment at a tertiary-level outpatient clinic. Participants with a mini-mental state examination score ≥27, independence in instrumental activities of daily living, and no diagnosis of mild cognitive impairment were assessed for SMCs. Sociodemographic characteristics, comorbidities, and medication use data were also collected. A logistic regression model was employed to identify independent predictors of SMCs.

**Results:**

The median age of the participants was 76 years, and 64% were female. The prevalence of SMCs was 61.0%. Participants with SMCs had a lower educational level than those without SMCs, with median (interquartile range, 25–75) values of 5 (5–12) and 5 (5–16) years, respectively. Attainment of a university-level education was independently associated with a significantly lower likelihood of reporting SMCs (OR = 0.262, 95% CI: 0.116–0.588, p = 0.001). No other variables were significantly associated with the risk of SMCs after adjustment for covariates.

**Conclusion:**

SMCs were highly prevalent among older adults in Türkiye. A university-level education was a significant protective factor against SMCs. Further prospective studies are warranted to gain a deeper understanding of the long-term effects of education level and other contributing factors on SMCs.

## 1. Introduction

Subjective memory complaints (SMCs) refer to self-perceived declines in cognitive functioning and are currently recognized as a core diagnostic criterion for identifying mild cognitive impairment (MCI) [1,2]. These complaints may be reported by individuals themselves or by informants familiar with their cognitive functioning. SMCs provide a practical means of capturing everyday cognitive challenges that may go undetected by standardized neuropsychological assessments.

SMCs are frequently observed among older adults, with prevalence estimates in community-dwelling populations ranging from 25% to 50% [3]. Epidemiological evidence indicates that SMCs are more prevalent among females and are associated with multimorbidity, depressive symptoms, polypharmacy, chronic pain, lower self-rated health, impaired instrumental activities of daily living (IADLs), and socioeconomic adversity [4–6]. Additionally, individuals with mild traumatic brain injury may report SMCs more frequently [6].

Research interest in SMCs has increased due to their potential role as early indicators of cognitive decline and subsequent progression to dementia. Beyond their prognostic significance, SMCs have been linked to reduced functional capacity, an increased risk of institutionalization, and poorer mental health outcomes [7–10].

A systematic review of 16 studies found that older adults with SMCs have a 1.5- to 3-fold higher risk of developing MCI or dementia than those without such complaints [11]. Longitudinal data indicate that 24.4% of individuals with SMCs progress to MCI, and 10.9% subsequently develop dementia [12]. These findings underscore the clinical relevance of SMCs in identifying individuals at elevated risk, thereby enabling early intervention and improved outcomes.

In Türkiye, as in many other countries, the prevalence of dementia has been increasing in parallel with population aging [13,14]. However, due to sociocultural factors such as patriarchal norms and delayed healthcare-seeking behavior among older adults, cognitive symptoms are often misattributed to the normal aging process.

In recent years, SMCs have gained recognition as potential early indicators of neurodegenerative processes. Several studies have explored the clinical correlates of SMCs in Türkiye [15,16]. However, no epidemiological studies have examined the prevalence of SMCs among cognitively intact, community-dwelling adults aged ≥65 years in Türkiye.

Early detection and management of SMCs are essential for mitigating the progression and overall burden of cognitive decline. Therefore, this study aimed to estimate the prevalence of SMCs among older adults and to identify associated demographic and clinical factors.

## 2. Materials and methods

**Figure 1:**
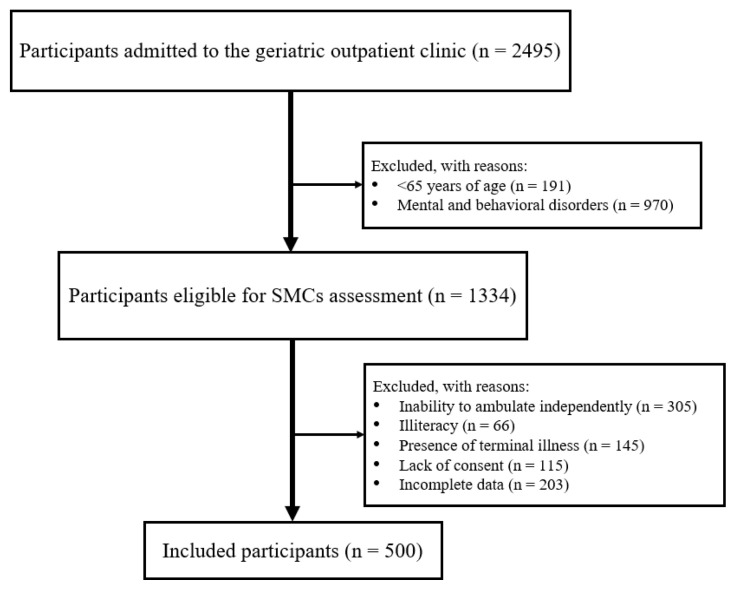
Flow diagram of the sample selection process. SMCs: subjective memory complaints.

In this retrospective, cross-sectional, observational study, 500 community-dwelling individuals aged 65 years or older were selected from a cohort of 2495 participants who had undergone a comprehensive geriatric assessment at a tertiary-level geriatric outpatient clinic. Exclusion criteria included neurocognitive and behavioral disorders (such as MCI, dementia, delirium, active affective disorders, psychotic disorders, and intellectual disability); inability to ambulate independently; moderate to severe hearing or visual impairments unresponsive to treatment; terminal illness; and refusal or lack of consent to participate in the study. A total of 1995 individuals (79.8%) from the initial cohort were excluded based on predefined eligibility criteria. Figure 1 illustrates the process of forming the study population.

Prior to enrollment, all participants provided written informed consent. The study was approved by the Institutional Ethics Committee and conducted in accordance with the ethical principles of the Declaration of Helsinki (project no. 19/404).

The prevalence of SMCs among community-dwelling older adults has been reported as 45.2% [17]. Based on a 95% confidence interval (CI) and a 5% margin of error, the required sample size was calculated as 381 participants.

### 2.1. Assessment of subjective memory complaints

Cognitive function in all participants was evaluated using the mini-mental state examination (MMSE), a standardized and widely used instrument in clinical practice [18,19]. Functional capacity related to complex, independent living skills was assessed using the IADL scale, which evaluates an individual’s ability to perform higher-level daily tasks [20]. This scale assesses functional independence across multiple domains, including telephone use, shopping, meal preparation, house cleaning, laundry, transportation, medication management, and financial handling. In its original form, the scale differentiates between male and female participants, reflecting traditional sex roles at the time of its development. Female participants were assessed in all eight domains. Male participants were evaluated in five domains, with meal preparation, housekeeping, and laundry excluded from the assessment [20]. MCI was identified according to the criteria proposed by Petersen et al. [21]. These criteria included: (1) the presence of memory complaints, (2) preserved ability to perform daily living activities, (3) overall intact cognitive functioning, (4) objectively measured memory impairment relative to age, and (5) absence of a dementia diagnosis. Participants who met all of these criteria were classified as having MCI and were excluded from the study [21].

Participants who scored ≥27 on the MMSE, were independent in IADL, and did not meet the criteria for MCI were evaluated for SMCs using the following question: “Do you think your memory is poorer than that of other people of a similar age?” Individuals who responded “yes” to this question were classified as having SMCs, whereas those who responded “no” were categorized as not having SMCs. This question is one of the items included in the subjective memory complaints questionnaire [22]. The use of a simple binary response format facilitates consistent interpretation between participants and evaluators, thereby enhancing the validity of the data collected from community-dwelling older adults [23].

### 2.2. Data collection and measures

Data were obtained from participants and electronic medical records during outpatient clinic visits. For each participant, demographic and clinical characteristics were assessed, including demographic variables (date of birth, sex, educational level, and marital status) and comorbidities (hypertension, diabetes mellitus, cardiovascular disease (CVD), chronic obstructive pulmonary disease (COPD), chronic kidney disease (CKD), cerebrovascular disease, depression, insomnia, urinary incontinence, anemia, vitamin B_12_ deficiency, and hearing aid use). CKD is defined as structural or functional renal impairment or a persistently reduced estimated glomerular filtration rate below 60 mL/min/1.73 m^2^ lasting at least 3 months [24]. A hemoglobin concentration <13.0 g/dL in males and <12.0 g/dL in females was considered indicative of anemia [25]. Serum vitamin B_12_ levels <200 pg/mL were considered indicative of vitamin B_12_ deficiency [26]. Multimorbidity was defined as the coexistence of two or more chronic diseases in the same individual [27].

A medication history interview was also conducted. Individuals were considered to have polypharmacy if they were taking five or more medications simultaneously. Individuals were classified as having hyperpolypharmacy if they were taking 10 or more medications simultaneously [28]. Anticholinergic cognitive burden (ACB) was assessed using the anticholinergic cognitive burden scale [29]. The total ACB score for each participant was calculated by summing the assigned anticholinergic values of all pharmacological agents used during treatment. Participants were stratified into two groups according to their ACB scores: those with an ACB score of 0 (no anticholinergic exposure) and those with an ACB score ≥1 (anticholinergic exposure) [30]. The Charlson comorbidity index (CCI) was used to quantify the cumulative burden of chronic disease. Existing diseases identified at the time of outpatient admission were classified into 19 diagnostic categories included in the CCI scoring system. The sum of the corresponding points assigned to these diagnostic categories constituted the CCI score. Participants were further categorized into low-to-medium (0–2), high (3–4), and very high (≥5) groups according to the severity of their CCI scores [31,32].

### 2.3. Statistical analysis

Statistical analyses were performed using IBM SPSS Statistics software, version 25 (IBM Corp., Armonk, NY, USA). The normality of data distribution was assessed using the Kolmogorov–Smirnov and Shapiro–Wilk tests. Since the parametric variables did not follow a normal distribution, the Mann–Whitney U test was used for analysis. Descriptive statistics were presented as percentages for categorical variables and as medians with interquartile ranges (IQR, 25–75) for continuous variables. Comparative analyses of categorical variables were conducted using the chi square test to assess statistical significance between groups. A multivariate logistic regression model was used to examine the association between clinically relevant variables (age, sex, marital status, CCI, polypharmacy, and ACB), variables with p ≤ 0.05 in univariate analysis (education level), and the dependent variable (SMCs). Results were expressed as odds ratios (ORs) with 95% confidence intervals (CIs) to quantify the strength and precision of associations. Statistical significance was set at p < 0.05.

## 3. Results

Participant characteristics are presented in the Table. The median age was 76 years (IQR, 72–82 years). Among participants, 64% were female, and 14.4% were illiterate. The prevalence of SMCs was 61.0% (n = 305). Hypertension (73.2%), diabetes mellitus (39.2%), and CVD(32.6%) were the most common comorbidities. The majority of the sample had a high (55.6%) or very high (40.4%) comorbidity burden. Approximately one-third of the participants had polypharmacy (n = 199, 39.8%) and anticholinergic drug use (n = 184, 36.8%).

There was a statistically significant difference in education level between the SMC and non-SMC groups (p = 0.003; Table). The prevalence of SMCs was lower among individuals with a university education than among those with lower education (p < 0.001). Participants with SMCs had a lower educational level than those without SMCs, with median (IQR, 25–75) values of 5 (5–12) and 5 (5–16) years, respectively. No statistically significant differences were found between the groups for other variables (Table).

**Figure 2:**
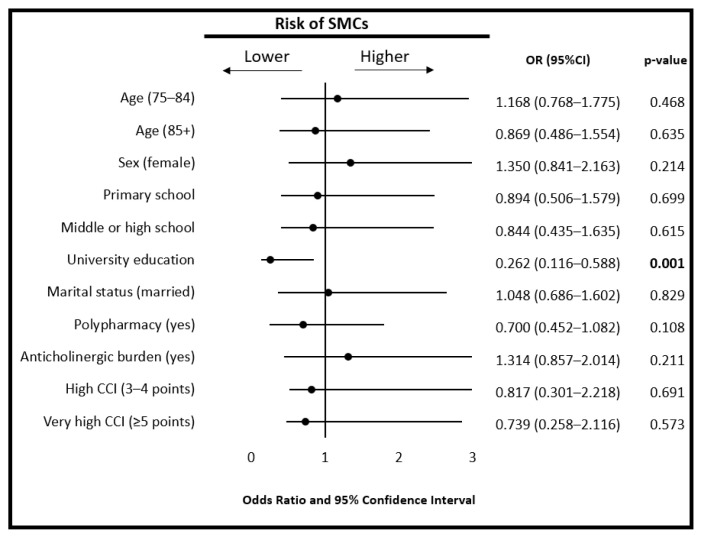
Forest plot showing the association between subjective memory complaints (SMCs) and demographic as well as comorbidity characteristics based on logistic regression analysis. SMCs: subjective memory complaints; CCI: Charlson comorbidity index.

A multivariate logistic regression analysis was performed to assess associations between SMCs and clinical variables. University-level education was associated with a significantly lower risk of SMCs (OR = 0.262, 95% CI: 0.116–0.588, p = 0.001). No other variables were significantly associated with the risk of SMCs after adjustment for covariates (Figure 2). The Hosmer–Lemeshow goodness-of-fit test indicated an adequate model fit (χ^2^ = 8.347, p = 0.400). The model explained 53.0% of the variance in SMCs (Nagelkerke R^2^).

## 4. Discussion

To the best of our knowledge, this is the first study conducted in Türkiye to investigate the prevalence and associated factors of SMCs among cognitively intact, community-dwelling adults aged 65 years and older. The prevalence of SMCs in our sample was 61%. A university-level education was identified as protective factor against SMCs. Several studies have linked high CCI and ACB scores to an increased risk of cognitive impairment [27], but our study did not find these factors to be statistically significant contributors to the higher prevalence of SMCs in this sample.

Given the growing burden of dementia, identifying individuals with SMCs who are at risk of progressing to cognitive impairment remains a key area of ongoing research. Community-based studies report a wide variation in the prevalence of SMCs, ranging from 10% to 60%, due to differences in study design, validity and reliability of measurement tools, and demographic characteristics of participants [3,33,34]. In our sample, the prevalence of SMCs was 61%, consistent with previous reports of 59% among community-dwelling older adults [35]. The study by Aysevener et al. was among the pioneering investigations on SMCs in Türkiye, reporting a prevalence of 42.5% [15]. However, our study differs methodologically by explicitly excluding individuals diagnosed with MCI, thereby allowing a more focused and homogeneous assessment of SMCs among cognitively normal older adults. The prevalence of memory complaints among older adults can vary considerably and is likely influenced by the specific questions asked and the characteristics of the surveyed population [36].

Our findings revealed a significant inverse relationship between educational attainment and the prevalence of SMCs. A recent study that partially supports our findings reported that individuals with higher education levels felt more satisfied with their memory and used more external strategies to support memory performance [37]. These findings suggest that formal education may play a significant role in enhancing cognitive abilities and improving the management of perceived memory difficulties. Furthermore, a recent metaanalysis suggested that higher education levels might serve as a protective factor, potentially delaying the transition from SMCs to MCI or dementia [38]. Collectively, these studies demonstrate that education exerts a crucial influence on memory perception and the future trajectory of cognitive functioning. Higher levels of education not only enhance cognitive reserve but also lead to beneficial alterations in brain structure and function, potentially reducing the likelihood of experiencing SMCs and the risk of developing neurodegenerative diseases [39]. These effects are likely attributable to education-driven physiological changes in brain plasticity, memory formation, and memory self-perception [40].

Several studies have demonstrated that multimorbidity adversely affects cognitive functioning [41–43]. Certain conditions, such as chronic pain, stroke, peptic ulcer disease, diabetes, CVD, and cancer, may be linked to memory complaints by adversely affecting brain health and thereby impairing cognitive function [44,45]. Similarly, the prevalence of SMCs tends to increase with a greater comorbidity burden [46]. However, our analysis revealed no significant association between SMCs and either specific disease patterns or the cumulative comorbidity burden. In other words, our results suggest that SMCs appear to be independent of specific morbidity patterns and overall comorbidity load. This finding contradicts previous research and may reflect the unique characteristics of our study sample. Our participants were community-dwelling, cognitively intact older adults with relatively high levels of independence and functional capacity, which may have mitigated the cognitive effects of physical illness. Additionally, differences in comorbidity assessment methods (e.g., self-report versus medical records), variations in sample size and age distribution, and inconsistent definitions of SMCs across studies may have contributed to these discrepancies. These factors underscore the complexity of disentangling the relationship between physical health and subjective cognitive experiences and emphasize the need for standardized methodologies in future research.

In our study, polypharmacy was observed in approximately half of the older adult population; however, no significant association was found between polypharmacy and the risk of SMCs. Interestingly, although the result did not reach statistical significance, the direction of the association suggested a lower likelihood of SMCs among individuals with polypharmacy. This finding contrasts with that of Veizi et al., who reported that polypharmacy was independently associated with an increased risk of SMCs, as well as with a higher anticholinergic burden and comorbidities such as COPD [47]. Similarly, another study reported that polypharmacy and a higher number of prescribed medications were more prevalent among patients with SMCs who subsequently developed dementia [48]. The divergence between our findings and previous evidence may reflect differences in study design, population characteristics, diagnostic criteria for SMCs, or overall comorbidity burden. It is also possible that individuals with polypharmacy in our cohort were under closer medical supervision, which may have facilitated better symptom recognition and management, thereby mitigating the risk of SMCs.

Several studies have supported the association between increased ACB and cognitive dysfunction [49–51]. In a study that followed 13,004 participants with and without cognitive dysfunction for 24 months, a greater decline in MMSE scores was observed among those using anticholinergic medications compared to nonusers [52]. In addition, previous studies have demonstrated that ACB increases the risk of dementia by more than fourfold among individuals with SMCs [48]. However, no significant association was observed between SMCs and ACB in the present study. The cross-sectional design of our study may have limited our ability to detect potential associations between these variables. This discrepancy from previous studies could be attributed to methodological differences, particularly the absence of temporal follow-up in our study design. Unlike our study, most research demonstrating significant associations between ACB and cognitive decline employed longitudinal designs, which are better suited for capturing cumulative or delayed effects over time. Larger sample sizes and longitudinal research are warranted to better elucidate the potential role of ACB in the development of SMCs.

Several limitations should be considered when interpreting our findings. First, although the sample size was statistically adequate based on power analysis, future studies with larger and more representative samples are needed to enhance the generalizability of the results. Second, a methodological limitation was the lack of a universally accepted and widely recognized standardized instrument for evaluating SMCs. Burmester et al. reported that different SMC assessment scales may yield significantly different results, with potentially important diagnostic and prognostic implications [39]. Finally, participants included in the study were required to score at least 27 out of 30 on the MMSE. However, this criterion may have excluded individuals who were unable to respond to certain items due to low literacy levels, visual impairments, or mild cognitive deficits, potentially introducing selection bias into the sample.

In conclusion, this study demonstrates that SMCs are highly prevalent among community-dwelling older adults in Türkiye, affecting more than half of the participants. Education level emerged as the only significant factor independently associated with SMCs, suggesting a potential protective effect of higher educational attainment. Given the high prevalence and potential implications of SMCs in predicting future cognitive decline, early identification—particularly among individuals with lower educational backgrounds—may facilitate timely interventions and monitoring strategies. Further longitudinal and population-based studies are warranted to validate these findings and to deepen our understanding of the pathways linking education, cognitive perception, and long-term cognitive outcomes in older adults.

## Figures and Tables

**Table:** Characteristics of the study population.

Variables	Total (n = 500)	Subjective memory complaints		p-value 1
		Yes (n = 305)	No (n = 195)	
Age (years) n (%)				
(65–74)	201 (40.2)	121 (39.7)	80 (41.0)	0.746
(75–84)	220 (44.0)	138 (45.2)	82 (42.1)	
(85+)	79 (15.8)	46 (15.1)	33 (16.9)	
Sex (female), n (%)	320 (64.0)	195 (63.9)	125 (64.1)	0.970
Education level, n (%)				
Illiterate	72 (14.4)	46 (15.0)	26 (13.3)	**0.003**
Primary school	235 (47.0)	149 (48.9)	86 (44.1)	
Middle or high school	136 (27.2)	88 (28.9)	48 (24.6)	
University education	57 (11.4)	22 (7.2)	35 (18.0)	
Marital status (married), n (%)	304 (60.8)	184 (60.3)	120 (61.5)	0.787
Hearing aid, n (%)	46 (9.2)	31 (10.2)	15 (7.7)	0.351
Hypertension, n (%)	366 (73.2)	224 (73.4)	142 (72.8)	0.878
Diabetes mellitus, n (%)	196 (39.2)	116 (38.0)	80 (41.0)	0.504
Cardiovascular disease, n (%)	163 (32.6)	100 (32.8)	63 (32.3)	0.911
COPD, n (%)	29 (5.8)	15 (4.9)	14 (7.2)	0.291
Chronic kidney disease, n (%)	132 (26.4)	78 (25.6)	54 (27.7)	0.600
Cerebrovascular disease, n (%)	12 (2.4)	6 (3.0)	6 (3.1)	0.936
Depression, n (%)	157 (31.4)	92 (30.2)	65 (33.3)	0.456
Insomnia, n (%)	79 (15.8)	43 (14.1)	36(18.5)	0.531
Urinary incontinence, n (%)	127 (25.4)	79 (25.9)	48 (24.6)	0.747
Anemia, n (%)	92 (18.4)	52 (17.0)	40 (20.5)	0.330
Vitamin B12 deficiency, n (%)	129 (25.8)	79 (25.9)	50 (25.6)	0.948
Multimorbidity, n (%)	388 (77.6)	232 (76.1)	156 (80.0)	0.303
Charlson comorbidity index, n (%)				
Low-to-medium	20 (4.0)	13 (4.3)	7 (3.6)	0.711
High	278 (55.6)	173 (56.7)	105 (53.8)	
Very high	202 (40.4)	119 (39.0)	83 (42.6)	
Polypharmacy, n (%)				
No	265 (53.0)	167 (54.8)	98 (50.2)	0.486
Yes	199 (39.8)	115 (37.7)	84 (43.1)	
Hyperpolypharmacy	36 (7.2)	23 (7.5)	13 (6.7)	
Anticholinergic burden (ACB) exposure, n (%)	184 (36.8)	116 (38.0)	68 (34.9)	0.475

1 Chi square test;

p < 0.05 considered statistically significant; COPD: chronic obstructive pulmonary disease.

## References

[1] Petersen RC Mild cognitive impairment as a diagnostic entity Journal of Internal Medicine 2004 256 3 183 194 10.1111/j.1365-2796.2004.01388.x 15324362

[2] Winblad B Palmer K Kivipelto M Jelic V Fratiglioni L Mild cognitive impairment-beyond controversies, towards a consensus: report of the International Working Group on Mild Cognitive Impairment Journal of Internal Medicine 2004 256 3 240 246 10.1111/j.1365-2796.2004.01380.x 15324367

[3] Jonker C Geerlings MI Schmand BA Are memory complaints predictive for dementia? A review of clinical and population-based studies International Journal of Geriatric Psychiatry 2000 15 11 983 991 10.1002/1099-1166(200011)15:11<983::AID-GPS238>3.0.CO;2-5 11113976

[4] Pinho PJMR Bertola L Ramos AA Ghossain Barbosa M Rabelo W Subjective memory complaints: Prevalence, associated factors and sex differences in the ELSI-Brazil study International Journal of Geriatric Psychiatry 2023 38 11 e6026 10.1002/gps.6026 37937726

[5] Meyer OL Leggett A Liu S Nguyen NH Prevalence and correlates of subjective memory complaints in Vietnamese adults International Psychogeriatrics 2018 30 7 1039 1048 10.1017/S104161021700254X 29198252 PMC5986585

[6] McAllister TW Saykin AJ Flashman LA Sparling MB Johnson SC Brain activation during working memory 1 month after mild traumatic brain injury: a functional MRI study Neurology 1999 53 6 1300 1308 10.1212/WNL.53.6.1300 10522888

[7] Crane MK Bogner HR Brown GK Gallo JJ The link between depressive symptoms, negative cognitive bias and memory complaints in older adults Aging and Mental Health 2007 11 6 708 715 10.1080/13607860701368497 18074258 PMC2825049

[8] Johansson B Allen-Burge R Zarit SH Self-reports on memory functioning in a longitudinal study of the oldest old: relation to current, prospective, and retrospective performance The Journals of Gerontology: Series B, Psychological Sciences and Social Sciences 1997 52 3 139 146 10.1093/geronb/52B.3.P139 9158565

[9] Pearman A Storandt M Predictors of Subjective Memory in Older Adults The Journals of Gerontology: Series B, Psychological Sciences and Social Sciences 2004 59 1 4 6 10.1093/geronb/59.1.P4 14722332

[10] Waldorff FB Siersma V Vogel A Waldemar G Subjective memory complaints in general practice predicts future dementia: a 4-year follow-up study International Journal of Geriatric Psychiatry 2012 27 11 1180 1188 10.1002/gps.3765 22253004

[11] Mendonça MD Alves L Bugalho P From Subjective Cognitive Complaints to Dementia: Who is at Risk?: A Systematic Review American Journal of Alzheimer’s Disease and Other Dementias 2016 31 2 105 114 10.1177/1533317515592331 PMC10852868 26142292

[12] Mitchell AJ Beaumont H Ferguson D Yadegarfar M Stubbs B Risk of dementia and mild cognitive impairment in older people with subjective memory complaints: meta-analysis Acta Psychiatrica Scandinavica 2014 130 6 439 451 10.1111/acps.12336 25219393

[13] Gurvit H Emre M Tinaz S Bilgic B Hanagasi H The prevalence of dementia in an urban Turkish population American Journal of Alzheimer’s Disease and Other Dementias 2008 23 1 67 76 10.1177/1533317507310570 PMC10846186 18276959

[14] Keskinoğlu P Yaka E Uçku R Yener G Kurt P Prevalence and risk factors of dementia among community dwelling elderly people in Izmir, Turkey Turkish Journal of Geriatrics 2013 16 2 135 141

[15] Aysevener EO Direk N Onat Özsoydan E Diriöz M Relationship between subjective memory complaints and objective memory impairment in a community-dwelling elderly population Journal of Clinical Psychiatry 2018 21 4 334 340 10.5505/kpd.2018.95967

[16] Açıkgöz M Özen Baru TB Emre U Taşçılar N Atalay A Assessment of Relation Between Subjectıve Memory Complaınts and Objective Cognitive Performance of Elderly Over 55 Years Old Age Archives of Neuropsychiatry 2014 51 1 57 62 10.4274/npa.y6719 28360596 PMC5370255

[17] Menon J Kantipudi SJ Vinoth S Kuchipudi J Prevalence of subjective cognitive decline and its association with physical health problems among urban community dwelling elderly population in South India Alzheimer’s and Dementia 2025 21 2 e14505 10.1002/alz.14505 PMC11848155 39935341

[18] Güngen C Ertan T Eker E Yaşar R Engin F Reliability and validity of the standardized Mini Mental State Examination in the diagnosis of mild dementia in Turkish population Turkish Journal of Psychiatry 2002 13 4 273 281 12794644

[19] Babacan-Yıldız G Ur-Özçelik E Kolukısa M Işık AT Gürsoy E Validity and reliability studies of modified mini mental state examination (MMSE-E) for Turkish illiterate patients with diagnosis of Alzheimer disease Turkish Journal of Psychiatry 2016 27 1 41 46 27369684

[20] Lawton MP Brody EM Assessment of older people: self-maintaining and instrumental activities of daily living Gerontologist 1969 9 3 179 186 10.1093/geront/9.3_Part_1.179 5349366

[21] Petersen RC Smith GE Waring SC Ivnik RJ Tangalos EG Mild cognitive impairment: clinical characterization and outcome Archives of Neurology 1999 56 3 303 308 10.1001/archneur.56.3.303 10190820

[22] Özel Kızıl ET Duman B Altıntaş O Kırıcı S Bastug G Investigation of the psychometric properties of the Turkish form of subjective memory complaints questionnaire Turkish Journal of Geriatrics 2013 16 2 150 154

[23] Choe YM Byun MS Lee JH Sohn BK Lee DY Subjective memory complaint as a useful tool for the early detection of Alzheimer’s disease Neuropsychiatric Disease and Treatment 2018 14 2451 2460 10.2147/NDT.S174517 30288043 PMC6161713

[24] Chapter 1: Definition and classification of CKD Kidney International Supplements 2013 3 1 19 62 10.1038/kisup.2012.64 25018975 PMC4089693

[25] Cappellini MD Motta I Anemia in Clinical Practice-Definition and Classification: Does Hemoglobin Change With Aging? Seminars in Hematology 2015 52 4 261 269 10.1053/j.seminhematol.2015.07.006 26404438

[26] Allen LH How common is vitamin B-12 deficiency? The American Journal of Clinical Nutrition 2009 89 2 693S 696S 10.3945/ajcn.2008.26947A 19116323

[27] Skou ST Mair FS Fortin M Guthrie B Nunes BP Multimorbidity Nature Reviews Disease Primers 2022 8 1 48 10.1038/s41572-022-00376-4 PMC7613517 35835758

[28] Nishtala PS Salahudeen MS Temporal Trends in polypharmacy and hyperpolypharmacy in older New Zealanders over a 9-Year Period: 2005–2013 Gerontology 2015 61 3 195 202 10.1159/000368191 25428287

[29] Boustani M Campbell N Munger S Maidment I Fox C Impact of anticholinergics on the aging brain: A review and practical application Aging Health 2008 4 3 311 320 10.2217/1745509X.4.3.311

[30] Lisibach A Benelli V Ceppi MG Waldner-Knogler K Csajka C Quality of anticholinergic burden scales and their impact on clinical outcomes: a systematic review European Journal of Clinical Pharmacology 2021 77 2 147 162 10.1007/s00228-020-02994-x 33011824 PMC7803697

[31] Charlson ME Pompei P Ales KL MacKenzie CR A new method of classifying prognostic comorbidity in longitudinal studies: Development and validation Journal of Chronic Diseases 1987 40 5 373 383 10.1016/0021-9681(87)90171-8 3558716

[32] Charlson M Szatrowski TP Peterson J Gold J Validation of a combined comorbidity index Journal of Clinical Epidemiology 1994 47 11 1245 1251 10.1016/0895-4356(94)90129-5 7722560

[33] Geerlings MI Jonker C Bouter LM Adèr HJ Schmand B Association between memory complaints and incident Alzheimer’s disease in elderly people with normal baseline cognition American Journal of Psychiatry 1999 156 4 531 537 10.1176/ajp.156.4.531 10200730

[34] Jorm AF Christensen H Henderson AS Korten AE Mackinnon AJ Complaints of cognitive decline in the elderly: a comparison of reports by subjects and informants in a community survey Psychological Medicine 1994 24 2 365 374 10.1017/S0033291700027343 8084932

[35] Blazer DG Hays JC Fillenbaum GG Gold DT Memory complaint as a predictor of cognitive decline: a comparison of African American and White elders Journal of Aging and Health 1997 9 2 171 184 10.1177/089826439700900202 10182402

[36] Rönnlund M Sundström A Adolfsson R Nilsson LG Subjective memory impairment in older adults predicts future dementia independent of baseline memory performance: Evidence from the Betula prospective cohort study Alzheimer’s and Dementia 2015 11 11 1385 1392 10.1016/j.jalz.2014.11.006 25667997

[37] Csábi E Hallgató E Volosin M The association between metamemory, subjective memory complaints, mood, and well-being: the Hungarian validation of Multifactorial Memory Questionnaire Cognitive Research: Principles and Implications 2023 8 1 15 10.1186/s41235-023-00469-y 36786909 PMC9928992

[38] Arora S Patten SB Mallo SC Lojo-Seoane C Felpete A The influence of education in predicting conversion from subjective cognitive decline (SCD) to objective cognitive impairment: A systematic review and meta-analysis Ageing Research Reviews 2024 101 102487 10.1016/j.arr.2024.102487 39243892

[39] Burmester B Leathem J Merrick P Assessing subjective memory complaints: a comparison of spontaneous reports and structured questionnaire methods International Psychogeriatrics 2015 27 1 61 77 10.1017/S1041610214001161 24989800

[40] Gonzalez-Gomez R Legaz A Moguilner S Cruzat J Hernández H Educational disparities in brain health and dementia across Latin America and the United States Alzheimer’s and Dementia 2024 20 9 5912 5925 10.1002/alz.14085 PMC11497666 39136296

[41] Zhang Y Yuan X Jiang Z Hu R Liang H The relationship between multimorbidity and cognitive function in older Chinese adults: based on propensity score matching Frontiers in Public Health 2024 12 1422000 10.3389/fpubh.2024.1422000 39328989 PMC11425792

[42] AlHarkan KS Aldhawyan AF Bahamdan AS Alqurashi YD Aldulijan FA Association between multimorbidity and cognitive decline in the elderly population of the Eastern Province, Saudi Arabia Journal of Family and Community Medicine 2024 31 2 99 106 10.4103/jfcm.jfcm_268_23 38800794 PMC11114873

[43] Scarmeas N Luchsinger JA Schupf N Brickman AM Cosentino S Physical activity, diet, and risk of Alzheimer disease Journal of the American Medical Association 2009 302 6 627 637 10.1001/jama.2009.1144 19671904 PMC2765045

[44] Sachs-Ericsson N Joiner T Blazer DG The influence of lifetime depression on self-reported memory and cognitive problems: results from the National Comorbidity Survey-Replication Aging and Mental Health 2008 12 2 183 192 10.1080/13607860801951739 18389398

[45] Comijs HC Kriegsman DM Dik MG Deeg DJ Jonker C Somatic chronic diseases and 6-year change in cognitive functioning among older persons Archives of Gerontology and Geriatrics 2009 48 2 191 196 10.1016/j.archger.2008.01.005 18299158

[46] Aarts S van den Akker M Hajema KJ van Ingen AM Metsemakers JFM Multimorbidity and its relation to subjective memory complaints in a large general population of older adults International Psychogeriatrics 2011 23 4 616 624 10.1017/S1041610210002024 21044401

[47] Yavuz Veizi BG Oktay Oğuz E Naharci MI Subjective Memory Complaints in Older Adults: The Role of Polypharmacy and Anticholinergic Burden Journal of Geriatric Psychiatry and Neurology 2025 0 0 10.1177/08919887251339837 40312279

[48] Naharci MI Cintosun U Ozturk A Oztin H Turker T Effect of anticholinergic burden on the development of dementia in older adults with subjective cognitive decline Psychiatry and Clinical Psychopharmacology 2017 27 3 263 270 10.1080/24750573.2017.1358130

[49] Pasina L Djade CD Lucca U Nobili A Tettamanti M Association of anticholinergic burden with cognitive and functional status in a cohort of hospitalized elderly: comparison of the anticholinergic cognitive burden scale and anticholinergic risk scale: results from the REPOSI study Drugs and Aging 2013 30 2 103 112 10.1007/s40266-012-0044-x 23239364

[50] Campbell NL Boustani MA Lane KA Gao S Hendrie H Use of anticholinergics and the risk of cognitive impairment in an African American population Neurology 2010 75 2 152 159 10.1212/WNL.0b013e3181e7f2ab 20625168 PMC2905930

[51] Shah RC Janos AL Kline JE Yu L Leurgans SU Cognitive decline in older persons initiating anticholinergic medications PLoS One 2013 8 5 e64111 10.1371/journal.pone.0064111 23741303 PMC3669362

[52] Fox C Richardson K Maidment ID Savva GM Matthews FE Anticholinergic medication use and cognitive impairment in the older population: the medical research council cognitive function and ageing study Journal of the American Geriatrics Society 2011 59 8 1477 1483 10.1111/j.1532-5415.2011.03491.x 21707557

